# Evaluating [^225^Ac]Ac-FAPI-46 for the treatment of soft-tissue sarcoma in mice

**DOI:** 10.1007/s00259-024-06809-4

**Published:** 2024-07-15

**Authors:** Marco F. Taddio, Suraj Doshi, Marwan Masri, Pauline Jeanjean, Firas Hikmat, Alana Gerlach, Lea Nyiranshuti, Ethan W. Rosser, Dorthe Schaue, Elie Besserer-Offroy, Giuseppe Carlucci, Caius G. Radu, Johannes Czernin, Katharina Lückerath, Christine E. Mona

**Affiliations:** 1 Department of Molecular and Medical Pharmacology, David Geffen School of Medicine at UCLA, Los Angeles, CA USA; 2 Department of Radiation Oncology, David Geffen School of Medicine at UCLA, Los Angeles, CA USA; 3 Department of Nuclear Medicine, University of Duisburg-Essen, and German Cancer Consortium (DKTK)-University Hospital Essen, Essen, Germany

**Keywords:** Actinium-225, Fibroblast activation protein, Sarcoma, FAPI-46, Theranostic, Immune checkpoint blockade

## Abstract

**Purpose:**

Fibroblast Activation Protein (FAP) is an emerging theranostic target that is highly expressed on cancer-associated fibroblasts and on certain tumor cells including sarcoma. We investigated the anti-tumor efficacy of [^225^Ac]Ac-FAPI-46 as monotherapy or in combination with immune checkpoint blockade (ICB) in immunocompetent murine models of sarcoma sensitive or resistant to ICB.

**Methods:**

[^68^Ga]Ga- and [^225^Ac]Ac-FAPI-46 were tested in subcutaneous FAP+ FSA fibrosarcoma bearing C3H/Sed/Kam mice. The efficacy of up to three cycles of 60 kBq [^225^Ac]Ac-FAPI-46 was evaluated as monotherapy and in combination with an anti-PD-1 antibody. Efficacy of [^225^Ac]Ac-FAPI-46 and/or ICB was further compared in FAP-overexpressing FSA (FSA-F) tumors that were sensitive to ICB or rendered ICB-resistant by tumor-induction in the presence of Abatacept.

**Results:**

[^225^Ac]Ac-FAPI-46 was well tolerated up to 3 × 60 kBq but had minimal effect on FSA tumor growth. The combination of three cycles [^225^Ac]Ac-FAPI-46 and ICB resulted in growth delay in 55% of mice (6/11) and partial tumor regression in 18% (2/11) of mice. In FSA-F tumors with FAP overexpression, both [^225^Ac]Ac-FAPI-46 and ICB were effective without additional benefits from the combination. In locally immunosuppressed and ICB resistant FAP-F tumors, however, [^225^Ac]Ac-FAPI-46 restored responsiveness to ICB, resulting in significant tumor regression and tumor-free survival of 56% of mice in the combination group up to 60 days post treatment.

**Conclusion:**

[^225^Ac]Ac-FAPI-46 efficacy is correlated with tumoral FAP expression levels and can restore responsiveness to PD-1 ICB. These data illustrate that careful patient selection based on target expression and rationally designed combination therapies are critically important to maximize the therapeutic impact of FAP-targeting radioligands.

**Supplementary Information:**

The online version contains supplementary material available at 10.1007/s00259-024-06809-4.

## Introduction

The peptidomimetic FAPI-46 targets fibroblast activation protein (FAP) and can be labeled with diagnostic or therapeutic radioisotopes for theranostic applications [1, 2]. While high levels of FAP are primarily found on cancer-associated fibroblasts (CAFs), some cancer cells such as sarcomas can also express substantial amounts of FAP. Among 28 different cancer types, [^68^Ga]Ga-FAPI uptake and target-to-background ratios were highest in sarcoma [3]. An association between tumoral [^68^Ga]Ga-FAPI uptake by positron emission tomography (PET) and histopathologic FAP expression has been observed in sarcoma patients [4, 5]. Due to their broad applicability and highly specific tumor uptake [2, 6], FAP-targeting molecules are being investigated as theranostic agents in various tumors. In patients with metastasized sarcoma, radioligand therapy (RLT) with [^90^Y]Y-FAPI-46 was well-tolerated and resulted in disease control in 7/12 patients (stable disease *n* = 6, partial response *n* = 1) [7]. FAP-targeting radioligands with longer tumor retention and radiotherapeutic strategies leading to higher tumor radiation doses may be required to enhance the efficacy of FAP-RLT in sarcoma.

In the current study, we use a syngeneic model of murine fibrosarcoma (FSA), which expresses FAP, is radiosensitive and amenable to immunotherapeutic perturbation [8]. We hypothesize that FSA tumors show limited efficacy to FAPI-46 RLT monotherapy due to the fast pharmacokinetics of FAPI-46 [2] resulting in a short tumor retention time and limited radiation dose deposition [9]. We demonstrate that FAP-RLT can be rendered more efficacious by either *(i)* repeat FAP-RLT cycles and/or *(ii)* higher target expression. FAP may have immunosuppressive effects in the tumor microenvironment (TME) [8, 10–13], which could be enhanced by the upregulation of programmed death-ligand 1 (PD-L1) expression on cancer cells in response to radiation [14] or exposure to interferon-g (IFNγ) secreted by T cells [15], which can suppress T cell activation and as a consequence attenuate RLT efficacy. We show that these effects can be mitigated and even exploited by combining RLT with PD-1 immune checkpoint blockade (ICB). In addition, our data suggest that FAP-RLT can restore responsiveness to programmed cell death protein 1 (PD-1) ICB in immunologically cold tumors.

## Materials and methods

### Cells

Fibrosarcoma cells (FSA) were a gift from the Department of Radiation Oncology at UCLA originally isolated from a methylcholanthrene-induced fibrosarcoma in C3Hf/He (C3H/Sed/Kam) mice [16]. Human embryonic kidney (HEK) 293T cells were obtained from the American Type Culture Collection. Cells were maintained in Dulbecco’s Modified Eagle Medium with 10% fetal bovine serum (Omega Scientific, Tarzana, CA, USA) at 37ºC and 5% CO_2_. To generate cells overexpressing murine FAP (mFAP), FSA cells were transduced with the lentiviral vector pLV-mFAP and dilution cloning was performed to select clones with low (FSA-F_low_), medium (FSA-F_med_) and high (FSA-F_hi_) levels of mFAP (Supplemental Fig. 1). Cell media was tested every 2–4 weeks for mycoplasma contamination using MycoAlert (LT07-710, Lonza, Basel, Switzerland).

### In vitro expression levels of FAP, PD-L1 and H-2 K (MHC class I) in FSA cells

FSA cells were seeded in 6-well plates (3 × 10^5^ / well) and treated after 24 h with either 10 ng/mL murine interferon-g (IFNγ) or with 8 Gy X-rays using a Gulmay RS320 X-ray unit at 300 kV and 10 mA (Gulmay Medical Ltd., Surrey, UK). Dosimetry was based on a Capintec ionization chamber calibrated to NIST standards and film (GAFCHROMIC EBT2, International Specialty Products, Wayne, NJ, USA). Treated cells and controls were collected 24 h after incubation and analyzed by either flow cytometry or immunoblot.

To quantify cell surface protein expression, 0.5 × 10^6^ cells were stained for 30 min on ice with anti-FAP (clone 73.3, 1:1000, Sigma-Aldrich, St. Louis, MO, USA), anti-PD-L1-PE (clone 10 F.9G2, 1:200, BioLegend, San Diego, CA, USA) or anti-H-2 K (MHC class I; clone Y-3, 1:40, Sigma-Aldrich) antibodies. Anti-IgG1-APC (15 min on ice; clone RMG1-1, 1:200, BioLegend) or anti-mIgG-FITC (15 min on ice; clone H + L, 1:1000, Invitrogen, Waltham, MA, USA) antibodies were used as secondary antibody for the detection of FAP and H2-K, respectively. All samples were measured on a LSRII Flow Cytometer (BD, Franklin Lakes, NJ, USA) at the UCLA Flow Cytometry Core Facility and analyzed using FlowJo software (FlowJo LLC, Ashland, OR, USA).

For immunoblot analysis, protein lysates were prepared in cold RIPA buffer supplemented with protease and phosphatase inhibitors, normalized using BCA assay, resolved on 4–12% Bis-Tris gels and electro-transferred onto nitrocellulose membranes. After blocking with 5% nonfat milk in TBS + 0.1% Tween-20 (TBS-T), membranes were cut into relevant sections and incubated overnight with the respective primary antibody (vinculin: clone E1E9V, phospho-STAT1 Tyr701: clone 58D6, Cell Signaling Technology, Danvers, MA, USA) diluted 1:1000 in 5% BSA in TBS-T. Membranes were washed and incubated with HRP-linked secondary antibodies at 1:2500 dilution in 5% nonfat dry milk / TBS-T. HRP was activated by incubating membranes with a mixture of SuperSignal Pico and SuperSignal Femto ECL reagents (100:1 ratio, ThermoFisher, Waltham, MA, USA). Exposure of photo film was used for detection.

### [^68^Ga]Ga-FAPI-46

[^68^Ga]Ga-FAPI-46 was synthesized as previously described [1, 2]. The final product had a radiochemical purity of > 98% by HPLC and thin-layer chromatography with 50 mM EDTA as a mobile phase and a molar activity of 130 MBq/µmol.

### Cell binding and internalization studies

Cells were plated in 24-wells (7.5 × 10^4^ / well) and incubated for 24 h. Binding studies were performed as previously described [2]. Briefly, after washing twice with PBS, 20 kBq [^68^Ga]Ga-FAPI-46 were added and incubated for 1 h at 37ºC and 5% CO_2_. Surface bound fractions were collected with ice-cold 1 M glycine-HCl (pH 2.2) and cells were lyzed subsequently using two sequential washes with aqueous 0.3 M NaOH. Samples were counted on a PerkinElmer 2480 Wizard2 Automatic Gamma Counter (PerkinElmer, Waltham, MA, USA). Data were decay-corrected, background-subtracted and resulting activities were normalized to 10^5^ cells.

### Animal models

In vivo studies were approved by the UCLA Institutional Animal Care and Use Committee (#2005-090) and conducted in three murine models: (1) tumors derived from the inoculation of parental FSA, (2) FSA clones transduced with murine FAP (low/medium/high) and (3) locally immunosuppressed FSA-F_med_ tumors. For all cell lines, tumor growth was tested in syngeneic, immunocompetent C3H/Sed/Kam mice. Female 6–8 weeks old mice (Department of Radiation Oncology, UCLA) were housed under gnotobiotic conditions (12 h–12 h light-dark cycle; food and water ad libitum). For tumor cell transplantation, cells were trypsinized, filtered through a 70 μm cell strainer and washed twice with PBS (500 x g, 4 °C, 5 min). Cells (0.5 × 10^6^ per animal) were resuspended in ice-cold PBS and mixed with an equal amount of matrigel (Corning Inc, Corning, NY, USA) to a total injection volume of 100 µL per animal. To generate locally immunosuppressed tumors, FSA-F_med_ cells were co-inoculated with the immunosuppressive and human/mouse cross-reactive CTLA-4 fusion protein Abatacept [17], in order to block CD80/86-mediated co-stimulation of T cells in the TME and thereby render tumors non-responsive to PD-1 ICB. For this, the cell pellet was resuspended in cold PBS containing 10 mg/mL Abatacept (final concentration = 0.5 mg/inoculation) before mixing with matrigel. Cells were inoculated subcutaneously into the right shoulder region of mice. Tumors reached a volume of 50–100 mm^3^ within 10–14 days (FSA), approximately 18 days (FSA-F_med_), and 16 days (locally immunosuppressed FSA-F_med_).

### Micro positron emission tomography (PET)/computed tomography (CT)

For in vivo FAP imaging, animals were intravenously (i.v.) injected with 1.1 MBq [^68^Ga]Ga-FAPI-46. After 60 min, animals were anesthetized and kept under 2% isoflurane for static 10 min PET scans with subsequent CT. Mice underwent CT scans every 4–7 days to monitor tumor size. All scans were performed on a G8 benchtop PET/CT (SOFIE Biosciences, Culver City, CA, USA). OsiriXv.10.0.2 (Pixmeo, Bernex, Switzerland) [18] was used for PET and CT analysis. For analyzing PET data, the 3D ball isocontour function was used to define the maximal and mean standardized uptake values (SUV_max_, SUV_mean_) of tumors. CT data were analyzed by delineating tumors on ≥ 7 CT slices and using the compute volume function to derive tumor volumes.

### Immunohistochemistry (IHC)

Formalin-fixed paraffin-embedded tumor samples (4 μm) were stained with an anti-FAP antibody (EPR20021, 1:50, Abcam, Cambridge, UK) as described previously [19]. For staining of T cells, antigens were retrieved according to the manufacturer’s instructions and specimens were incubated overnight at 4 °C with either anti-CD4 (clone EPR19514, 1:1000, Abcam) or anti-CD8 (clone 4SM15, 1:500, ThermoFisher) antibodies. All samples were subsequently stained with DAB Chromogen reagent (#K3467, Dako, Glostrup, Denmark) and counterstained with hematoxylin before dehydration, mounting with Permount Mounting Media and digital scanning at 20x magnification using ScanScope AT (Leica Biosystems, Vista, CA, USA). Cell densities per area were estimated from the averaged CD4^+^ or CD8^+^ cell counts of five 200 × 200 μm squares (0.04 mm^3^).

### [^225^Ac]Ac-FAPI-46

FAPI-46 precursor was kindly provided by the University of Heidelberg and Actinium-225 was obtained from the National Isotope Development Center (Oak Ridge, TN, USA). Unless specified otherwise, all reagents and chemicals used were purchased from ThermoFisher Scientific and/or Sigma-Aldrich and used as received. For radiolabeling, [^225^Ac]Ac(NO_3_)_3_ was dissolved in 0.1 M HCl and mixed with FAPi-46 in 1 M NaOAc containing 10 mg/mL gentisic acid resulting in a final reaction pH of ~ 5.5 [20]. Incubation for thirty minutes at 90 °C provided [^225^Ac]Ac-FAPi-46 in a purity of 97 ± 2% and at a molar activity of 45–55 MBq/µmol. Final product stability was confirmed by TLC up to 24 h after labelling (98.5% intact).

### Therapy studies

For an initial activity escalation study, FSA-bearing mice were injected intravenously with 20, 40 or 60 kBq [^225^Ac]Ac-FAPi-46. For the combination study with one or three injections of 60 kBq [^225^Ac]Ac-FAPI-46 and anti-PD-1 therapy, FSA-bearing mice were treated with either a single injection of 60 kBq [^225^Ac]Ac-FAPI-46, or three consecutive injections of 60 kBq [^225^Ac]Ac-FAPI-46 in 24 h intervals. Anti-PD-1 antibody (intraperitoneal injection, 10 mg/kg in PBS; clone RMP1-14, #BE0146, BioXCell, Lebanon, NH, USA) or isotype-control (clone 2A3, #BE0089, BioXCell) treatment was started 24 h after the last [^225^Ac]Ac-FAPI-46 administration and injected every 3–4 days for a total of four doses.

Mice with locally immunosuppressed FSA-F_med_ tumors were treated with three consecutive injections of 60 kBq [^225^Ac]Ac-FAPI-46 in 24 h intervals. Anti-PD-1 antibody or isotype was administered as mentioned above.

For all studies, 8–12 animals were randomized to each group based on tumor volume, and tumor volumes as well as body weights were monitored by (semi-)weekly CT. Animals were euthanized when reaching a humane endpoint according to ARC protocol (tumors > 2cm^3^, ulceration) or a study endpoint (survival, tumor regrowth), and overall survival (OS) was calculated.

### Statistics

Unless stated otherwise, data are shown as mean ± SEM. Comparisons of two groups were evaluated using unpaired 2-tailed Student’s *t* test. Statistical analysis of more than two groups was performed using one-way analysis of variance (ANOVA) with Tukey’s post hoc correction. Median survival was analyzed using the log-rank test. *P* values of less than 0.05 were considered significant. GraphPad Prism software (version 9, GraphPad, San Diego, CA, USA) was used for all statistical calculations.

## Results

### FSA tumors express FAP

**Fig. 1:**
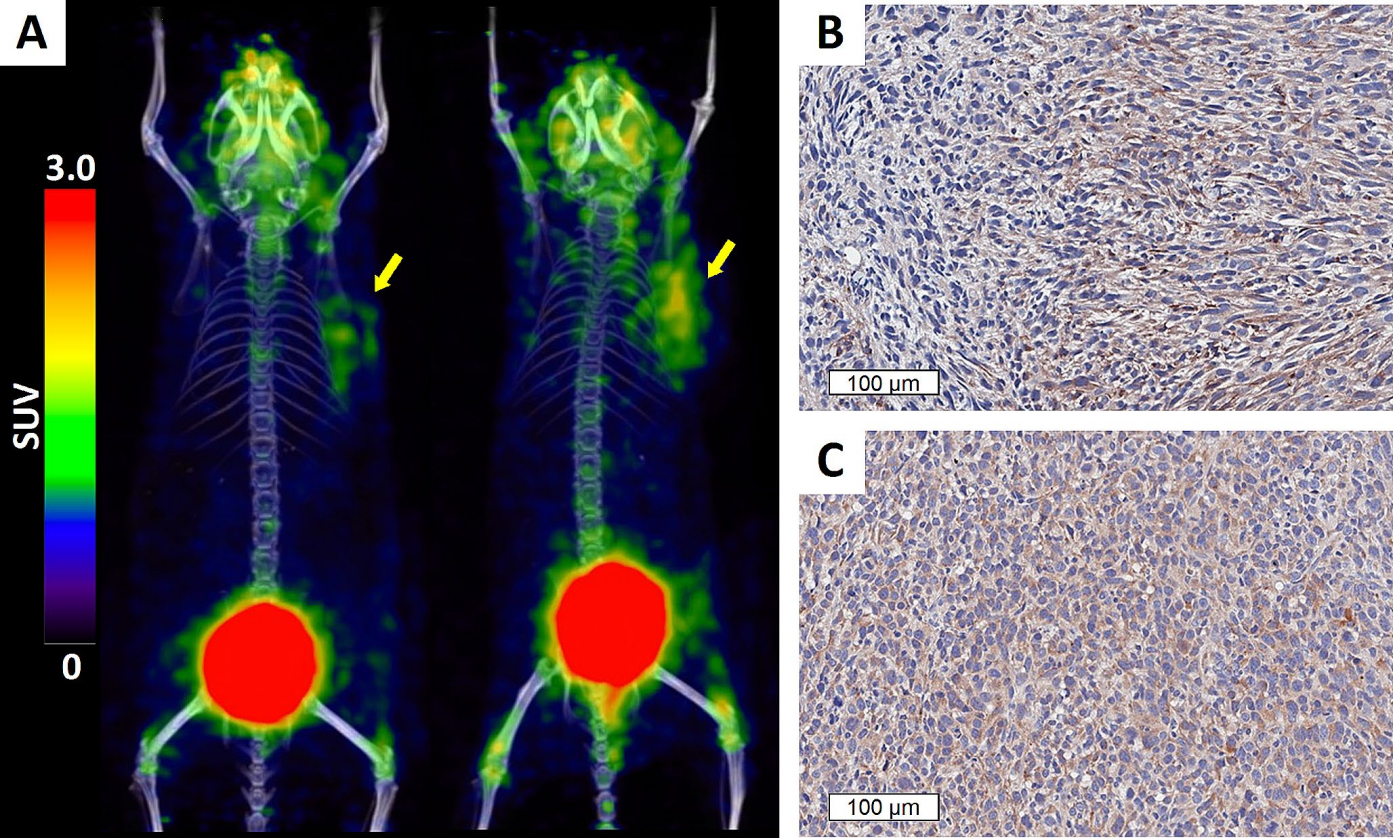
FAP expression in FSA tumors. (**A**) Maximal Intensity Projections (MIP) from PET/CT images of mice bearing FSA tumors 60 min after injection with 1.1 MBq [^68^Ga]Ga-FAPI-46 (2/13 mice are shown; left/right: SUV_max_ = 1.64/2.14, SUV_mean_ = 0.86/1.21, V = 290 mm^3^ (left) and 249 mm^3^ (right)). Yellow arrows, accumulation of [^68^Ga]Ga-FAPI-46 in tumor. (**B, C**) Representative staining (one tumor out of *n* = 3) of murine FAP immunoreactive cells in 4 μm tissue sections of 10d (**B**, one representative section out of three is shown) and 17d (**C**, *n* = 1) old FSA tumors show consistent and homogeneous FAP expression

FAP expression in FSA tumors was quantified using [^68^Ga]Ga-FAPI-46 PET/CT 11–19 days after tumor cell inoculation. SUV_max_ and SUV_mean_ of tumors were 1.55 ± 0.49 and of 0.91 ± 0.26, respectively (*n* = 13, Fig. 1A). Homogeneous intratumoral FAP expression was confirmed by IHC (Fig. 1B).

### FSA tumors are infiltrated with T cells and respond to IFNγ and radiation exposure

**Fig. 2:**
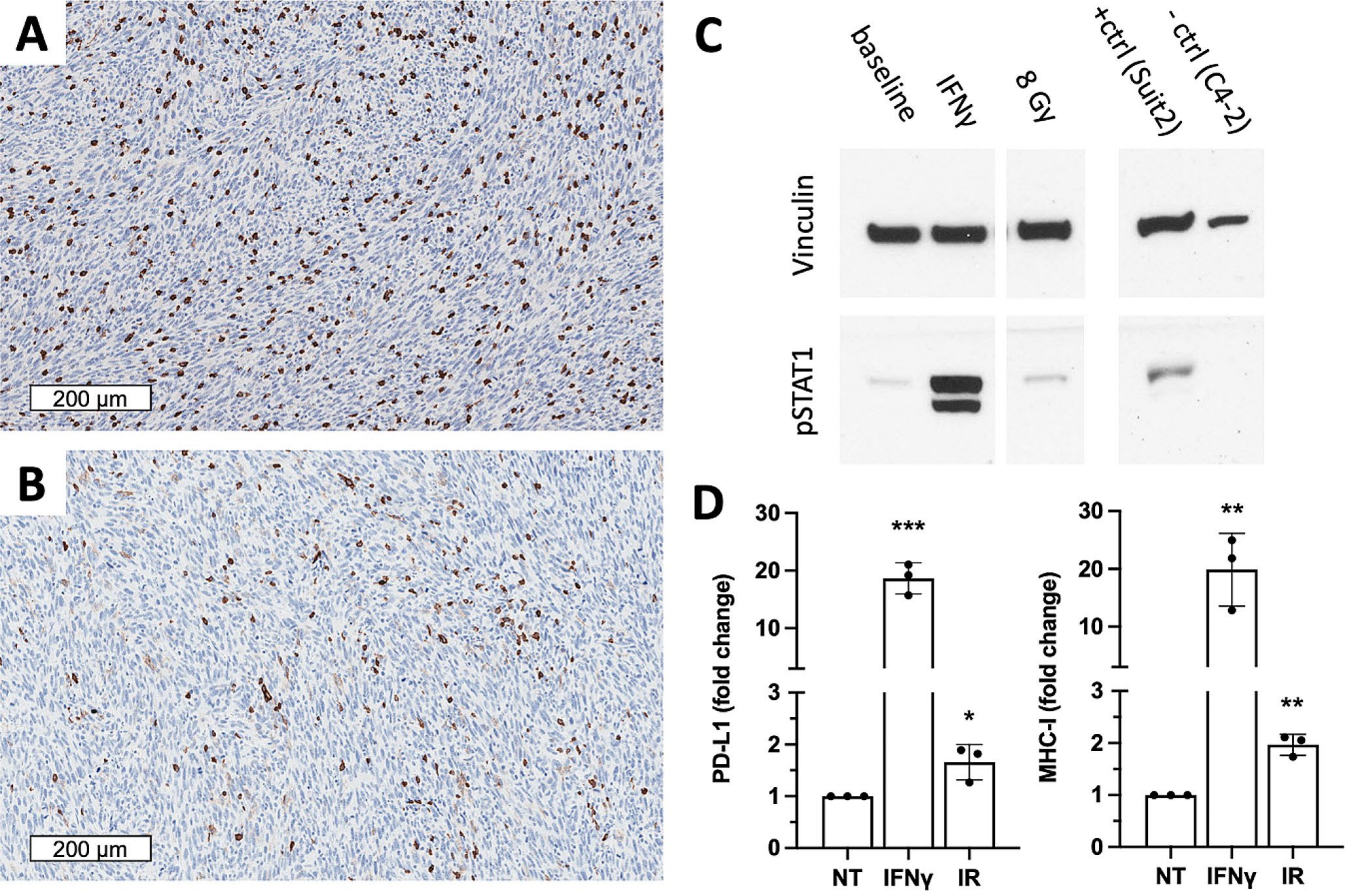
T cell infiltration, IFNγ sensing and radiation-induced PD-L1 and MHC-I expression suggest combination of FAP-RLT with PD-1 ICB. (**A**,**B**) Representative staining of FSA tumor sections (*n* = 4) for CD8^+^ (**A**) and CD4^+^ (**B**) T cells. (**C**) Immunoblot analysis of FSA cells 24 h after IFNγ treatment shows STAT1 phosphorylation. Treatments: murine IFNγ (10 ng/mL) or 8 Gy radiation (x-ray; IR). Controls: Suit2 cells (+), C4-2 cells (-). (**D**) Flow cytometric analysis of FSA cells (*n* = 3 per group; mean ± SD) 24 h after treatment with 10 ng/mL IFNγ or 8 Gy IR shows significant upregulation in PD-L1 and MHC-I expression. Data were normalized to untreated (NT) samples. *p < 0.05; **p < 0.005; ***p < 0.0005

To investigate the immune status of FSA tumors, infiltration of untreated FSA tumors with T cells was analyzed. Our results show substantial infiltration by CD8^+^ T cells (~ 555 cells/mm^2^; Fig. 2A) as well as CD4^+^ T cells (~ 330 cells/mm^2^; Fig. 2B). In addition, FSA cells treated with IFNγ in vitro activated STAT1 (Fig. 2C), and increased PD-L1 (18.7-fold), and MHC class I (19.9-fold) cell surface expression (Fig. 2D), confirming responsiveness of FSA cells to IFNγ. Likewise, irradiation of FSA cells increased PD-L1 and MHC class I expression (Fig. 2D), albeit to a lesser extent than following IFNγ exposure and independent of STAT1 phosphorylation. These results suggested that FSA tumors might respond to RLT by increasing expression of immunosuppressive cell surface antigens, a setting which could facilitate synergy between RLT and PD-1 ICB [21].

### Single activities of up to 60kBq [^225^Ac]Ac-FAPi-46 are well tolerated

**Fig. 3:**
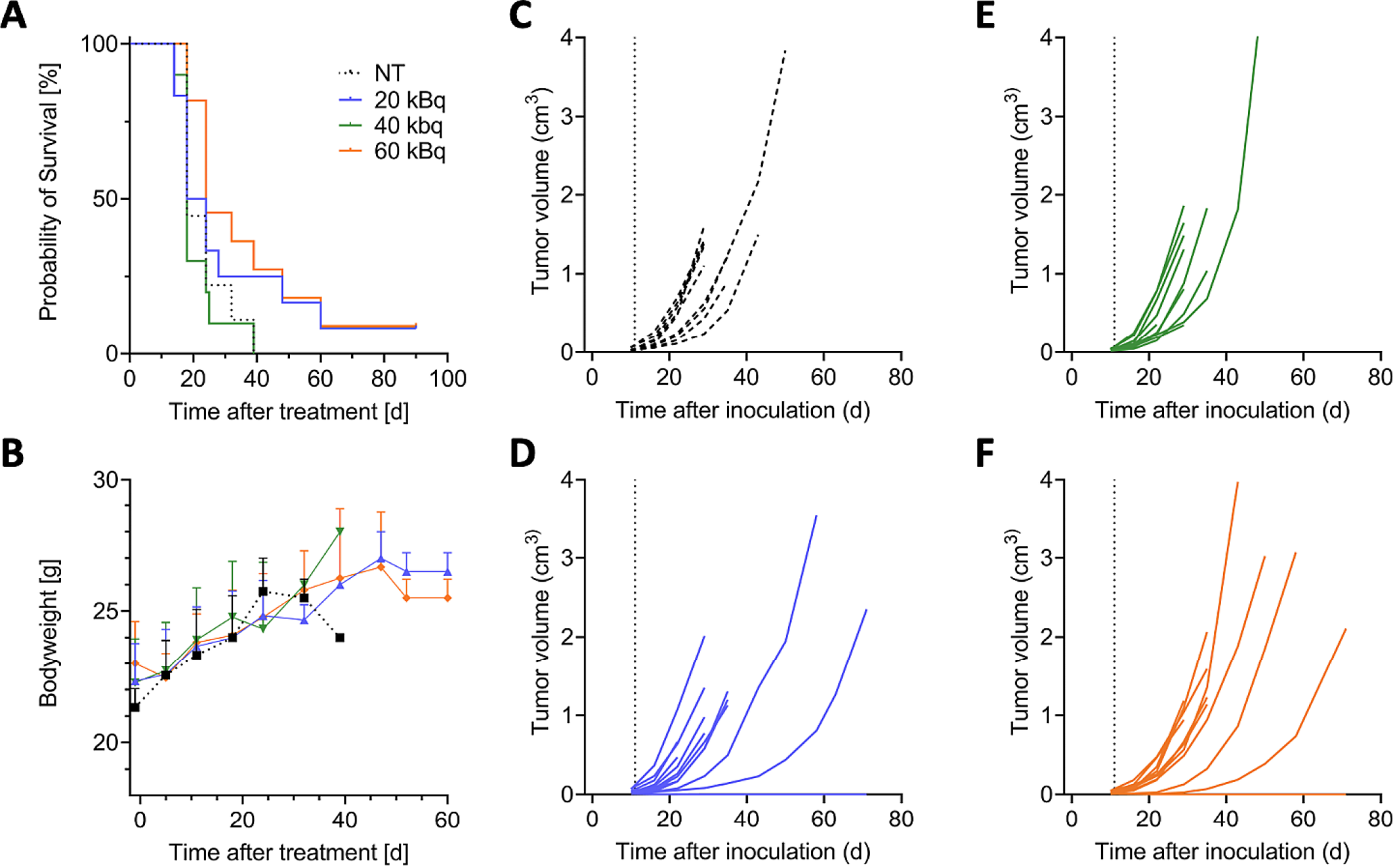
Activity escalation study of [^225^Ac]Ac-FAPi-46 in FSA tumor bearing C3H/Sed/Kam mice shows low efficacy for activity doses of up to 60 kBq. (**A**) Kaplan-Meier survival curves of untreated (NT, black, *n* = 9, 18d), 20 kBq (blue, *n* = 12, 18d, *p =* ns), 40 kBq (green, *n* = 11, 18d, *p =* ns) and 60 kBq (orange, *n* = 11, 24d, *p* = 0.0557) treated mice. (**B**) Development of animal bodyweights during study (mean±SD); no toxicity observed. (**C-F**) Individual tumor growth curves for untreated (**C**), 20 kBq (**D**), 40 kBq (**E**) and 60 kBq (**F**). Animals without tumor growth were not included for OS analysis. ns – nonsignificant

An initial activity escalation study was performed in FSA bearing C3H/Sed/Kam mice to test which injected activity is well tolerated and can achieve tumor growth retardation. Compared to untreated (NT) tumors (*n* = 9, OS = 29d), no survival benefit was observed following injection of 20 kBq (*n* = 12, *p =* 0.4543, OS = 32d) and 40 kBq (*n* = 11, *p =* 0.5767, OS = 29d); a trend in OS benefit was noted with 60 kBq [^225^Ac]Ac-FAPi-46 (*n* = 11, *p =* 0.0557, OS = 35d) (Fig. 3A). Activities up to 60 kBq were well tolerated as indicated by stable bodyweight (Fig. 3B) and caused tumor growth delay in some animals but without tumor regression (Fig. 3C-F).

### [^225^Ac]Ac-FAPI-46 combined with PD-1 ICB shows improved efficacy

**Fig. 4:**
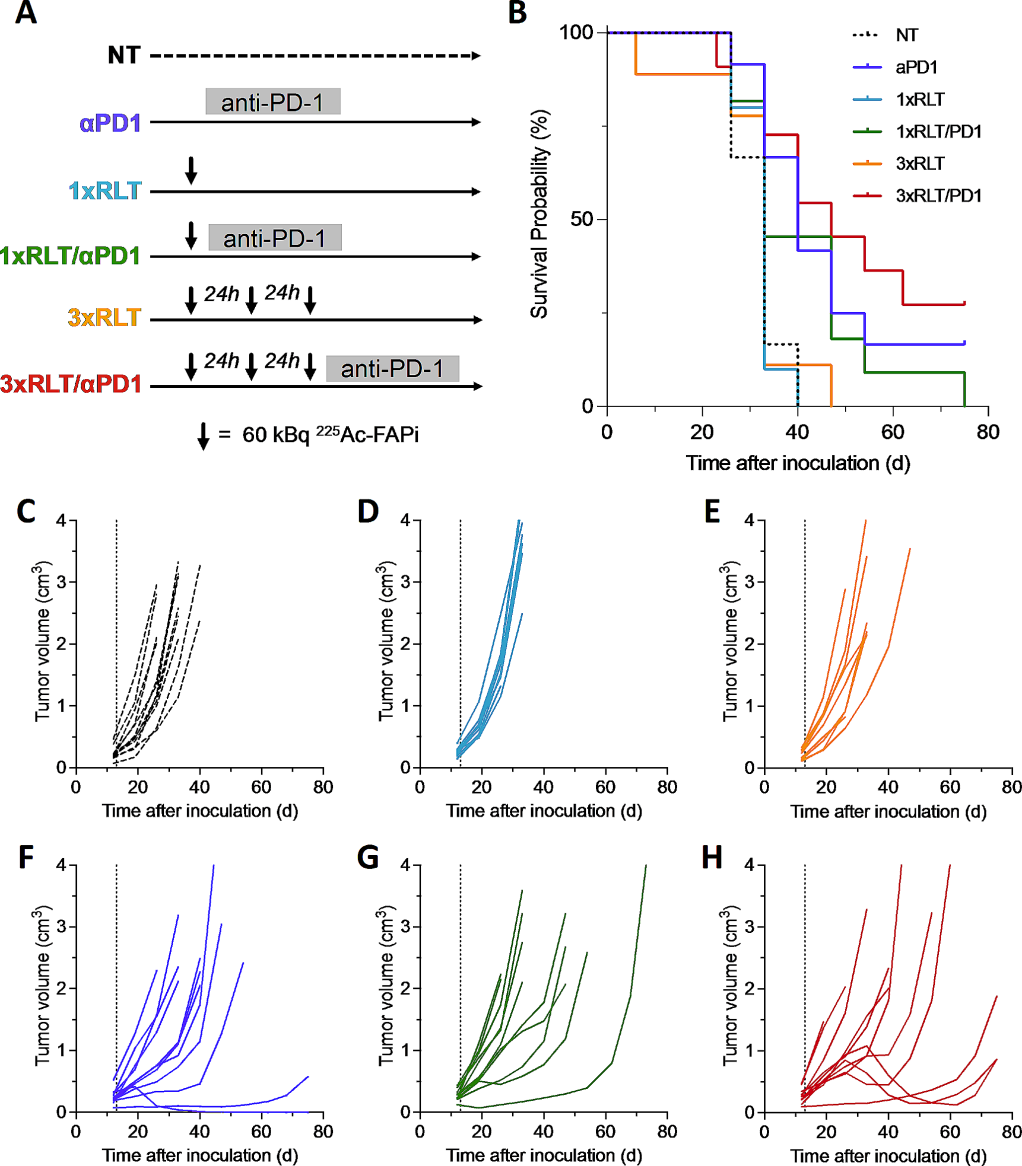
Combination of [^225^Ac]Ac-FAPI-46 with PD-1 ICB shows synergistic effects. (**A**) Study design. (**B**) Kaplan-Meier survival curves of untreated (*n* = 12, black, 33d), PD-1 ICB (*n* = 12, purple, 40d, *p* = 0.0041 vs. untreated), 1 × 60 kBq (*n* = 10, blue, 33d, *p* = 0.8649), 3 × 60 kBq (*n* = 9, orange, 33d, *p* = 0.9214), 1 × 60 kBq + PD-1 ICB (*n* = 11, green, 33d, *p* = 0.0664) and 3 × 60 kBq + PD-1 ICB (*n* = 11, red, 47d, *p* = 0.0051) treated mice. (**C-H**) Individual tumor growth curves for untreated (**C**), and 1 × 60 kBq [^225^Ac]Ac-FAPI-46 (**D**), 3 × 60 kBq [^225^Ac]Ac-FAPI-46 (**E**), PD-1 ICB (**F**), 1 × 60 kBq + PD-1 ICB (**G**) and 3 × 60 kBq + PD-1 ICB (**H**) treated mice. Dotted line, treatment start

Since injection of up to 60 kBq [^225^Ac]Ac-FAPI-46 was well-tolerated but did not result in significant anti-tumor effects, we increased the administration of [^225^Ac]Ac-FAPi-46 to three consecutive activities of 60 kBq in 24 h intervals (Fig. 4A) to increase the tumor radiation dose. The choice of intervals was based on the rapid pharmacokinetics and resulting short tumor retention of FAPi-46, with only 20% of the maximal tumor activity remaining after 24 h [2]. Triple injection of [^225^Ac]Ac-FAPI-46 did not improve survival compared to untreated animals or mice receiving a single injection of RLT (median survival for all groups, 33d) (Fig. 4B-E). Given the upregulation of PD-L1 on FSA cells in response to radiation (Fig. 2D), we tested if FAP-RLT could be rendered effective in combination with PD-1 ICB to harness RLT-induced immune activation (Fig. 4A). Treatment with PD-1 ICB alone caused significant tumor growth delay, increasing median survival from 33 days (untreated) to 40 days (*p* = 0.0041); the addition of a single cycle of [^225^Ac]Ac-FAPI-46 to ICB did not further improve outcome (*p =* 0.2864 versus PD-1 ICB) (Fig. 4B, F,G). However, three cycles FAP-RLT plus PD-1 ICB resulted in a prolonged median survival of 47 days (*p =* 0.0051 vs. untreated; *p =* 0.0095 vs. triple RLT; *p =* 0.5185 vs. PD-1 ICB) (Fig. 4B, H). Tumor growth was delayed in 6/11 animals, with temporary tumor regression in 2/11 animals, which relapsed on day 54 and 68 post treatment; in contrast, tumor growth slowed but tumors did not regress in animals treated with PD-1 ICB only.

### Increasing FAP tumor expression enhances tumor uptake

**Fig. 5:**
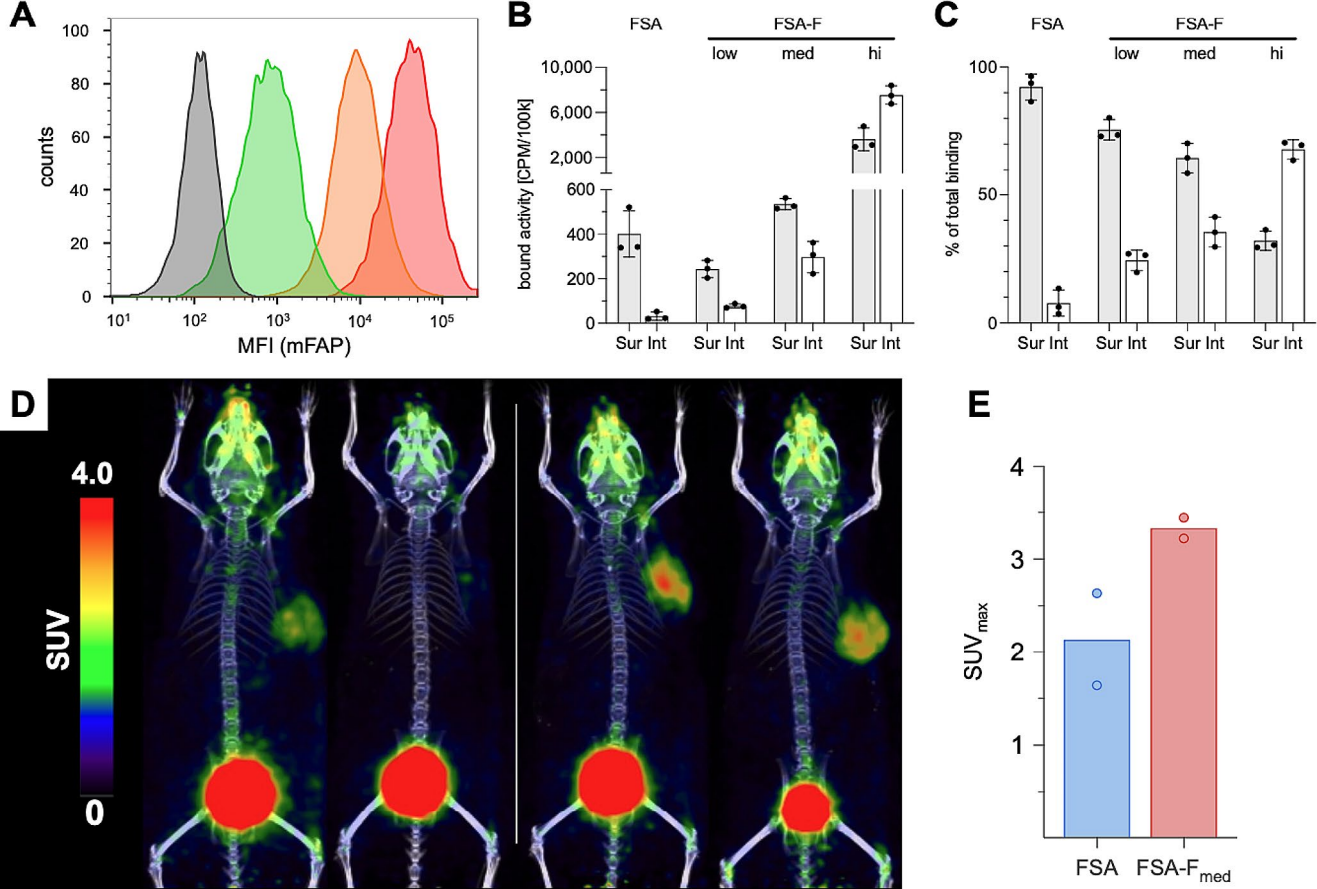
Internalization and [^68^Ga]Ga-FAPI-46 in vivo accumulation correlate positively with mFAP expression levels. (**A**) Representative flow cytometric histogram of mFAP cell surface levels in FSA (grey, MFI = 302 / unstained: not shown, MFI = 126), FSA-F_low_ (green, MFI = 1 082), FSA-F_med_ (orange, MFI = 11 586) and FSA-F_hi_ (red, MFI = 50 128) cells in vitro (1 × 10^5^ cells per sample; *n* = 3). (**B**) in vitro surface binding (Sur) and internalization (Int) in CPM per 10^5^ cells (*n* = 3/group; mean±SD) and (**C**) percentage of total binding and internalization (*n* = 3/group; mean±SD) of [^68^Ga]GaFAPi-46 in parental FSA cells and the three FSA-F cell lines. (**D**) MIP of [^68^Ga]Ga-FAPi-46 PET/CT scans of mice bearing FSA (left, *n* = 2) or FSA-F_med_ (right, *n* = 2) tumors (**E**) reaching SUV_max_ of 2.63/1.64 and 3.44/3.22, respectively

To address the limited efficacy of [^225^Ac]Ac-FAPI-46 observed in parental FSA tumors and to better recapitulate the high FAP expression in clinical sarcomas [19, 22], we generated FSA cells overexpressing low, medium and high levels of murine FAP (FSA-F). Flow cytometric analysis of in vitro cultured cells showed a mean fluorescence intensity (MFI) of 302 for parental FSA (only expressing FAP in vivo), whereas genetically modified FSA-F_low_, FSA-F_med_ and FSA-F_hi_ had substantially higher surface levels of FAP with MFIs of 1 082, 11 586 and 50 128, respectively (Fig. 5A). FAP expression levels correlated with specific [^68^Ga]GaFAPI-46 cell binding capacity, which increased from 321 ± 38 CPM/ 10^5^ cells for FSA-F_low_ to 832 ± 59 CPM/ 10^5^ cells for FSA-F_med_ and 11 167 ± 1 793 CPM/ 10^5^ cells for FSA-F_hi_ (Fig. 5B). Interestingly, higher FAP expression per cell was associated with higher radioligand internalization of up to 70% for FSA-F_hi_ and 36% for FSA-F_med_ compared to an average of 7.3% for parental FSA (Fig. 5C).

We then tested if FSA-F tumors can be established in syngeneic mice and retain FAP expression in vivo to investigate potential synergies of [^225^Ac]Ac-FAPI-46 with PD-1 ICB in a setting with high target expression. Test inoculation of FSA-F_low_, FSA-F_med_, and FSA-F_hi_ cells into C3H/Sed/Kam mice revealed high take rates for FSA-F_low_ (∼90%), lower take rates for FSA-F_med_ (∼75%) and no tumor growth for FSA-F_hi_ cells. FSA-F_med_ tumors were chosen for further investigations and stable in vivo FAP expression was confirmed by [^68^Ga]Ga-FAPI-46 PET/CT with SUV_max_ of 3.22 and 3.44, respectively (*n* = 2, Fig. 5D/E) up to at least 19d post inoculation.

### Increased [^225^Ac]Ac-FAPi-46 efficacy but no synergy with PD-1 ICB in the FSA-F_med_ model

**Fig. 6:**
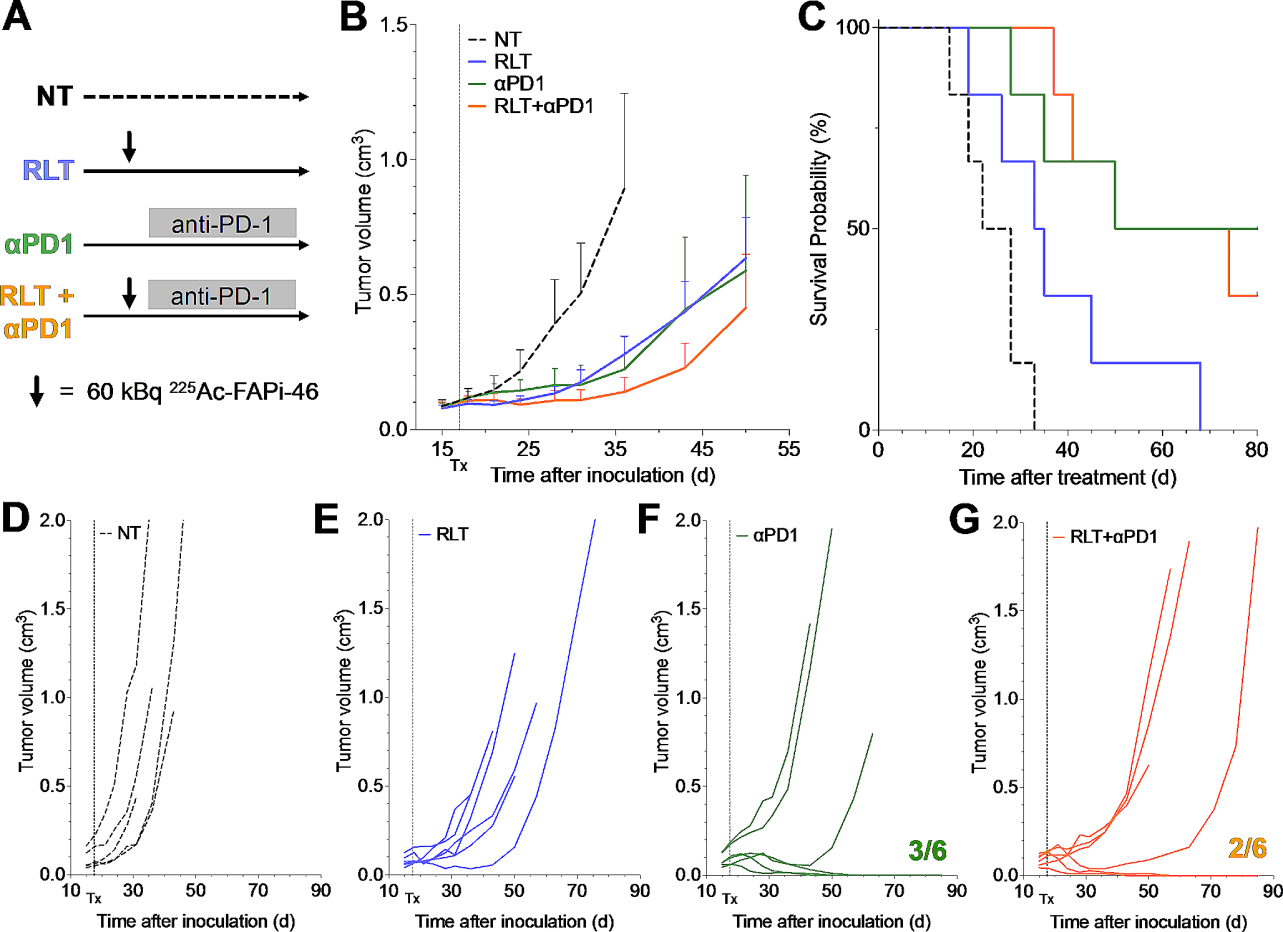
FSA-F_med_tumors respond to FAP-RLT. Efficacy study with untreated (NT, dashed, *n* = 5), 60 kBq [^225^Ac]Ac-FAPi-46 (RLT, blue, *n* = 6), PD-1 ICB (αPD1, green, *n* = 6) and combination (RLT + αPD1, yellow, *n* = 6) treated FSA-F_med_ tumor bearing C3H/Sed/Kam mice. (**A**) Study design; (**B**) Average tumor growth curves per group (mean ± SEM); (**C**) Kaplan-Meier survival curves with median survival for NT (dashed, 25d), RLT (blue, 34d, *p* = 0.0628 vs. NT), αPD1 (green, 52d, *p* = 0.0030 vs. NT) and RLT + αPD1 (yellow, 52d, *p =* 0.0006 vs. untreated; *p =* 0.8167 vs. PD-1 ICB); (**D-G**) Individual tumor growth curves for NT (**D**), 60kBq [^225^Ac]Ac-FAPi-46 (**E**), PD-1 ICB (**F**) and 60kBq [^225^Ac]Ac-FAPi-46 combined with PD-1 ICB (**G**). Dotted line, treatment start

While we showed that a single dose of 60 kBq [^225^Ac]Ac-FAPI-46 was inefficacious in the FSA tumor model, we tested RLT efficacy also in the FSA-F_med_ tumor bearing mice using 60 kBq [^225^Ac]Ac-FAPI-46, with or without PD-1 ICB (Fig. 6A). Increased target expression (> 50% higher SUV_max_ compared to parental FSA tumors) improved therapeutic efficacy of [^225^Ac]Ac-FAPI-46. However, the combination with PD-1 ICB did not further improve outcome compared to PD-1 ICB monotherapy, which may be the result of the already high efficacy of PD-1 ICB in this model (Fig. 6B-G).

### [^225^Ac]Ac-FAPI-46 restores PD-1 ICB efficacy in immunologically cold tumors

**Fig. 7:**
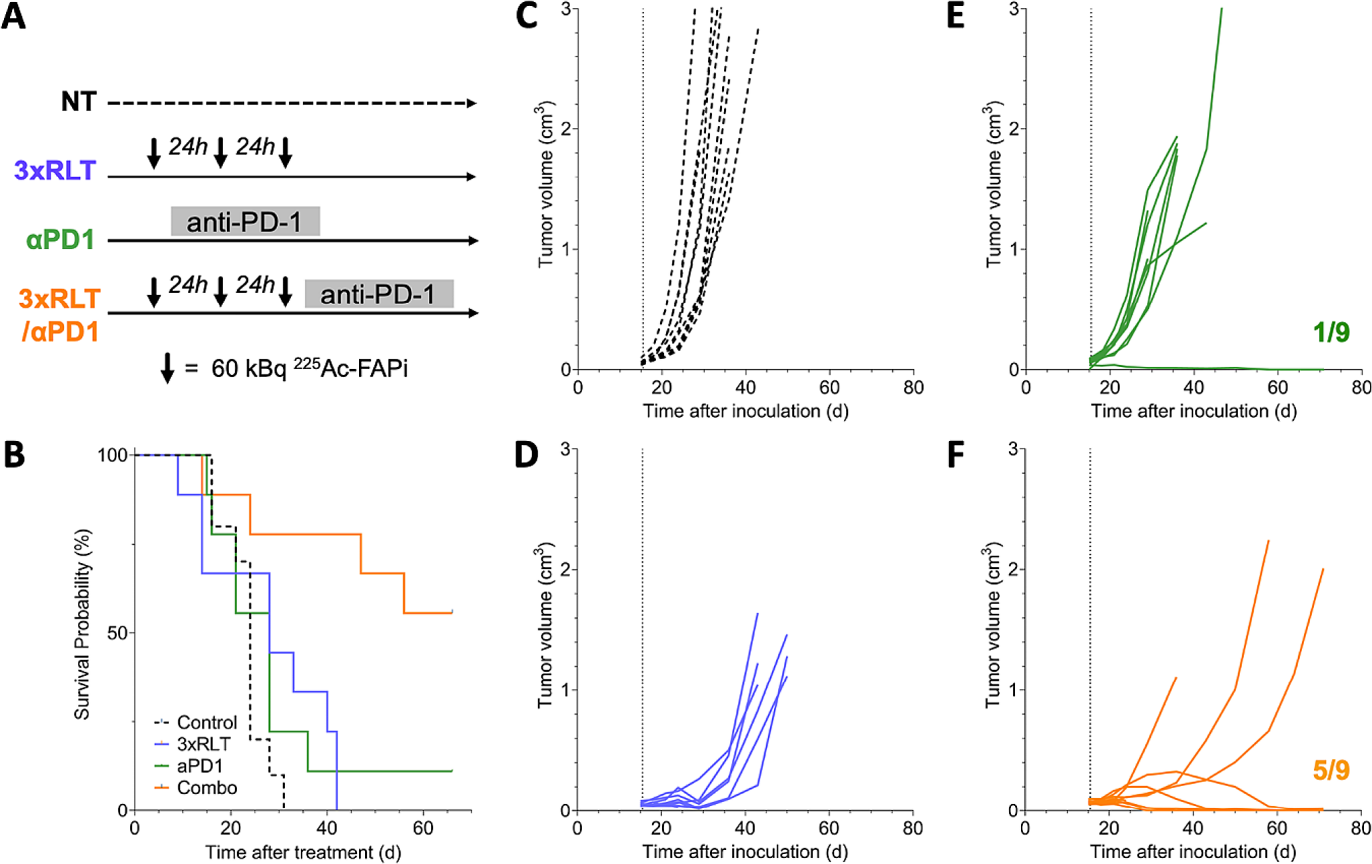
Combination of [^225^Ac]Ac-FAPi-46 with PD-1 ICB effectively overcomes an immunosuppressive TME. (**A**) Study design. (**B**) Kaplan-Meier survival curves of untreated (*n* = 10, black, 24d), and 3 × 60 kBq [^225^Ac]Ac-FAPi-46 (*n* = 9, blue, 28d, *p* = 0.083), PD-1 ICB (*n* = 9, green, 28d, *p* = 0.292) and 3 × 60 kBq + PD-1 ICB (*n* = 9, orange, OS not reached, *p* = 0.0015) treated mice. (**C-F**) Individual tumor growth curves for nontreated (**C**), 3 × 60 kBq [^225^Ac]Ac-FAPi-46 (**D**), PD-1 ICB (**E**), and 3 × 60 kBq + PD-1 ICB (**F**) treated mice. Dotted line, treatment start

In contrast to the FSA tumor model, human sarcoma generally exhibit rather low immunogenicity and clinical trials testing immunotherapy regiments in STS have only been modestly successful [23]. To test our approach in a more translationally relevant setting, we generated a model with attenuated PD-1 efficacy with the goal to investigate if FAP-RLT can render ICB effective when combined (Fig. 7A). Untreated animals reached a median survival of 24d, and neither treatment with 3 × 60 kBq [^225^Ac]Ac-FAPI-46 (28d, *p* = 0.083) nor with PD-1 ICB (28d, *p* = 0.292) improved survival (Fig. 7B). However, combining PD-1 ICB with [^225^Ac]Ac-FAPI-46 restored responsiveness to PD-1 ICB and resulted in significant tumor regression and tumor-free survival in 5/9 animals, which was sustained for at least up to 60 days post treatment (*p* = 0.0015 vs. NT; *p* = 0.0025 vs. NT; *p* = 0.029 vs. PD-1 ICB) (Fig. 7C-F).

## Discussion

To our knowledge, this is the first report of [^225^Ac]Ac-FAPI-46 RLT in a fully immunocompetent mouse model of fibrosarcoma with stable and homogenous expression of murine FAP, allowing the study of FAP-RLT and its combination with immunotherapeutic approaches. Until now, FAP-RLT has been tested preclinically in many immunocompromised xenograft models like human HT1080-FAP [24], HEK293-FAP and patient-derived sarcoma [25], as well as in human PANC-1 and MIA PaCa-2 xenografts [26, 27]. However, these models lack a fully functional immune system and, therefore, do not reflect the clinical situation. The importance of evaluating effects of FAP-RLT in an immunocompetent setting is further emphasized by recent findings of FAP as a potential predictive biomarker for PD-1 blockade in non-small cell lung cancer patients [28], which highlights the immunosuppressive role of FAP or FAP + cells.

By using FAPI-46 as a sub-efficacious FAP-RLT modality, due to its short tumor retention and fast clearance [2], we investigated the importance of essential parameters like target expression and tumor radiation dose for (FAP-)RLT efficacy. While increasing the total tumor radiation dose through repeated dosing with [^225^Ac]Ac-FAPI-46 resulted in limited tumor growth retardation in tumors with low FAP expression (parental FSA tumors), we show that higher FAP expression (FSA-F) correlates with improved FAP-RLT efficacy in immunocompetent animals. These results highlight the complex relation between target expression, tumor absorbed dose and dose fractionation strategies, and show a strong correlation of target expression with RLT efficacy [9], which underscores the importance of patient stratification for FAP-targeted RLT.

We and others have shown that irradiation induces immunosuppressive mechanisms like upregulation of PD-L1 on tumor cells, which can be successfully addressed with ICB. This is also supported by several ongoing clinical trials testing ICB in combination with external beam radiation therapy in sarcoma (e.g. NCT03116529, NCT03307616, NCT03474094). Our in vivo results reveal that the FSA and FSA-F_med_ tumor models are sensitive to PD-1 ICB monotherapy, but limited synergy was achieved by combining ICB with FAP-targeted RLT. To imitate the clinically lower responsiveness of sarcoma to anti-PD1 [29, 30], we induced local immunosuppression by co-inoculation of tumor cells with Abatacept. In this model, the anti-tumor effect of PD-1 ICB was heavily diminished. However, [^225^Ac]Ac-FAPI-46 restored responsiveness to PD-1 ICB and induced major synergistic effects, resulting in significant tumor regression and tumor-free survival in 56% of mice for at least 60 days post treatment. These results suggest that FAP-targeted RLT can increase immunogenicity in an immunosuppressed TME by re-sensitizing tumors to PD-1 ICB and emphasize the general need for suitable immunocompetent models to test RLT in combination with immunotherapeutic approaches.

Taken together, our results (1) support further development of FAP-RLT for the treatment of tumors with high FAP expression, (2) suggest that FAP-targeted RLT could be leveraged to re-sensitize immunosuppressed tumors for ICB, and (3) imply that the pharmacokinetic profile of FAPI-46 might be insufficient to achieve profound anti-tumor efficacy, which is why currently new compounds are being tested in clinical trials [31–34]. However, we also acknowledge that the chosen model with strong T cell infiltration and Abatacept co-inoculation is not fully reflective of the clinical situation in sarcoma. Future experiments will have to characterize this model further and investigate which RLT-related immunological effects can be translated to guide the rational design of novel FAP-RLT combination therapies with ICB in the clinics, while in parallel new immunocompetent mouse models reflecting immunologically cold sarcoma should be established. Overall, these results give hope that RLT could help to sensitize also other tumor types for immunotherapy.

## Electronic supplementary material

Below is the link to the electronic supplementary material.

Supplementary Material 2

Supplementary Material 3

Supplementary Material 4

Supplementary Material 5

## Data Availability

The datasets generated during and/or analysed during the current study are available from the corresponding author on reasonable request.
