# Supplementary material for: Evaluating [225Ac]Ac-FAPI-46 for the treatment of soft-tissue sarcoma in mice
Source: Eur J Nucl Med Mol Imaging. 2024 Jul 15;51(13):4026–37. doi: 10.1007/s00259-024-06809-4 (PMC11527918; doi:10.1007/s00259-024-06809-4)
Supplement: Supplementary file 1 — Supplementary Material 2 [file 259_2024_6809_MOESM2_ESM.docx]

# Evaluating [^225^Ac]Ac-FAPI-46 for the treatment of soft-tissue sarcoma in mice (Supplementary Information)

Marco F. Taddio, Suraj Doshi, Marwan Masri, Pauline Jeanjean, Firas Hikmat, Alana Gerlach, Lea Nyiranshuti, Ethan W. Rosser, Dorthe Schaue, Elie Besserer-Offroy, Giuseppe Carlucci, Caius G. Radu, Johannes Czernin, Katharina Lückerath, Christine E. Mona

**S1** – **Lentiviral Vector for mFAP transductions of FSA cells**

Transduction Methods: The lentiviral vector pLV-mFAP was generated using the backbone of pLV_mTurquoise2-iLID-CAAX (gift from Dr. Sean R Collins, Addgene plasmid # 160999). Replacement of mTurquoise2-ILID-CAAX sequence for mFAP was done by Genscript (Piscataway, NJ, USA). Briefly, mTurquoise2-ILID-CAAX sequence was removed by vector opening between BamHI and SmaI restrictions sites. The coding sequence of murine FAP (NM_007986.3) was synthetized with BamHI and SmaI overhangs cleavage sites at the 5’ and 3’ ends, respectively, and inserted into the opened vector backbone. Insertion was confirmed by Sanger sequencing of the complete vector. HEK293T cells were transiently co-transfected with the pLV-mFAP lentivector, pMD2.G (gift from Dr. Didier Trono, Addgene plasmid #12259) and psPAX2 (gift from Dr. Didier Trono, Addgene plasmid #12260) using Lipofectamine 3000 (ThermoFisher Scientific, Waltham, MA, USA). Viral particles were collected 48h post-transfection, filtered to remove any cell debris (0.45 μm PVDF syringe filter, ThermoFisher Scientific, Waltham, MA, USA) and used fresh to generate cells with stable mFAP expression. Dilution cloning was subsequently performed to achieve clones with mFAP expression resulting in cell lines FSA-F_low_ / FSA-F_med_ / FSA-F_hi_.

Plasmid map of the lentivector coding for murine FAP, pLV-mFAP.
