# Supplementary figures and images for: Evaluating [225Ac]Ac-FAPI-46 for the treatment of soft-tissue sarcoma in mice
Source: Eur J Nucl Med Mol Imaging. 2024 Jul 15;51(13):4026–37. doi: 10.1007/s00259-024-06809-4 (PMC11527918; doi:10.1007/s00259-024-06809-4)

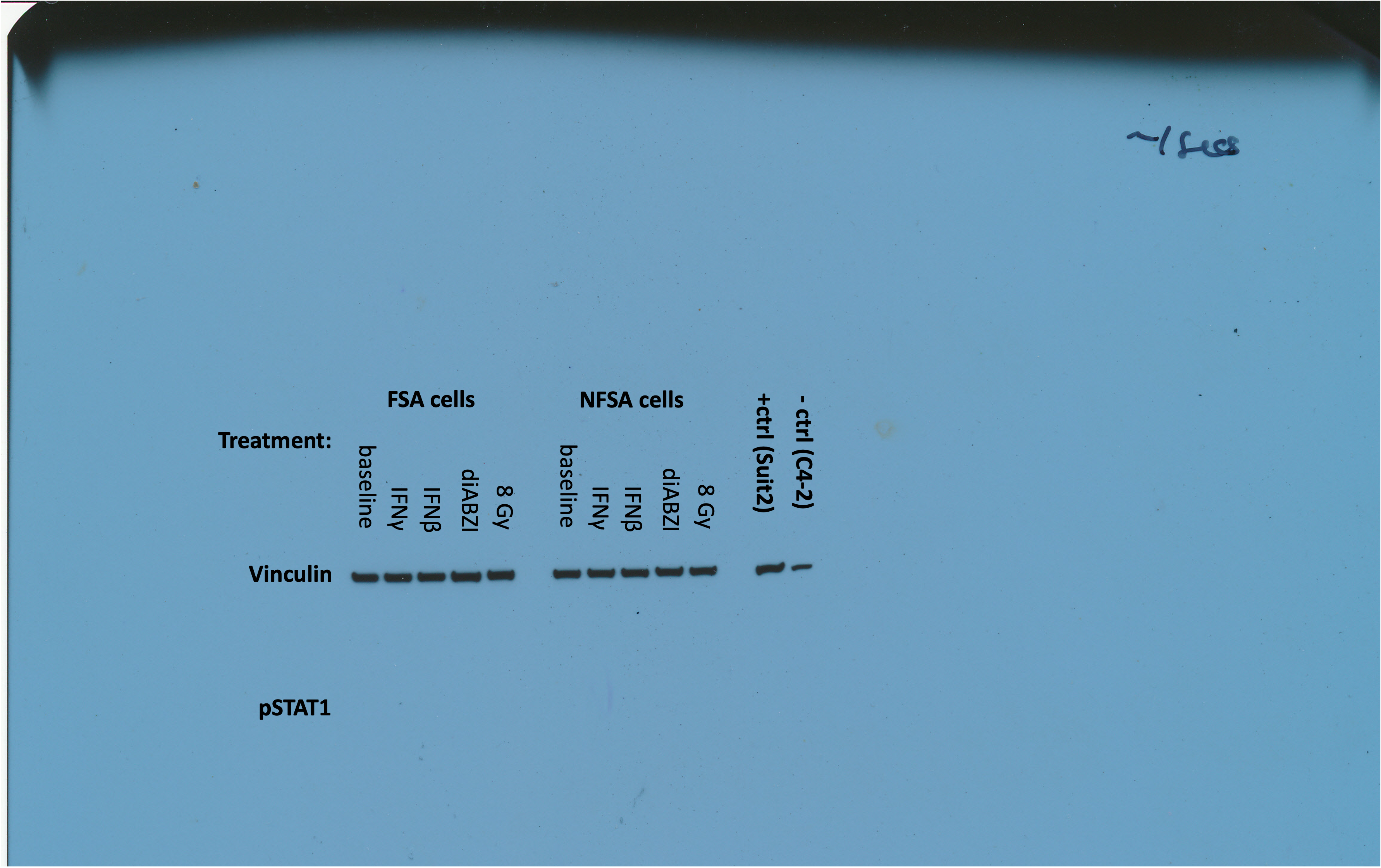

Supplement: Supplementary file 2 — Supplementary Material 3 [file 259_2024_6809_MOESM3_ESM.png]

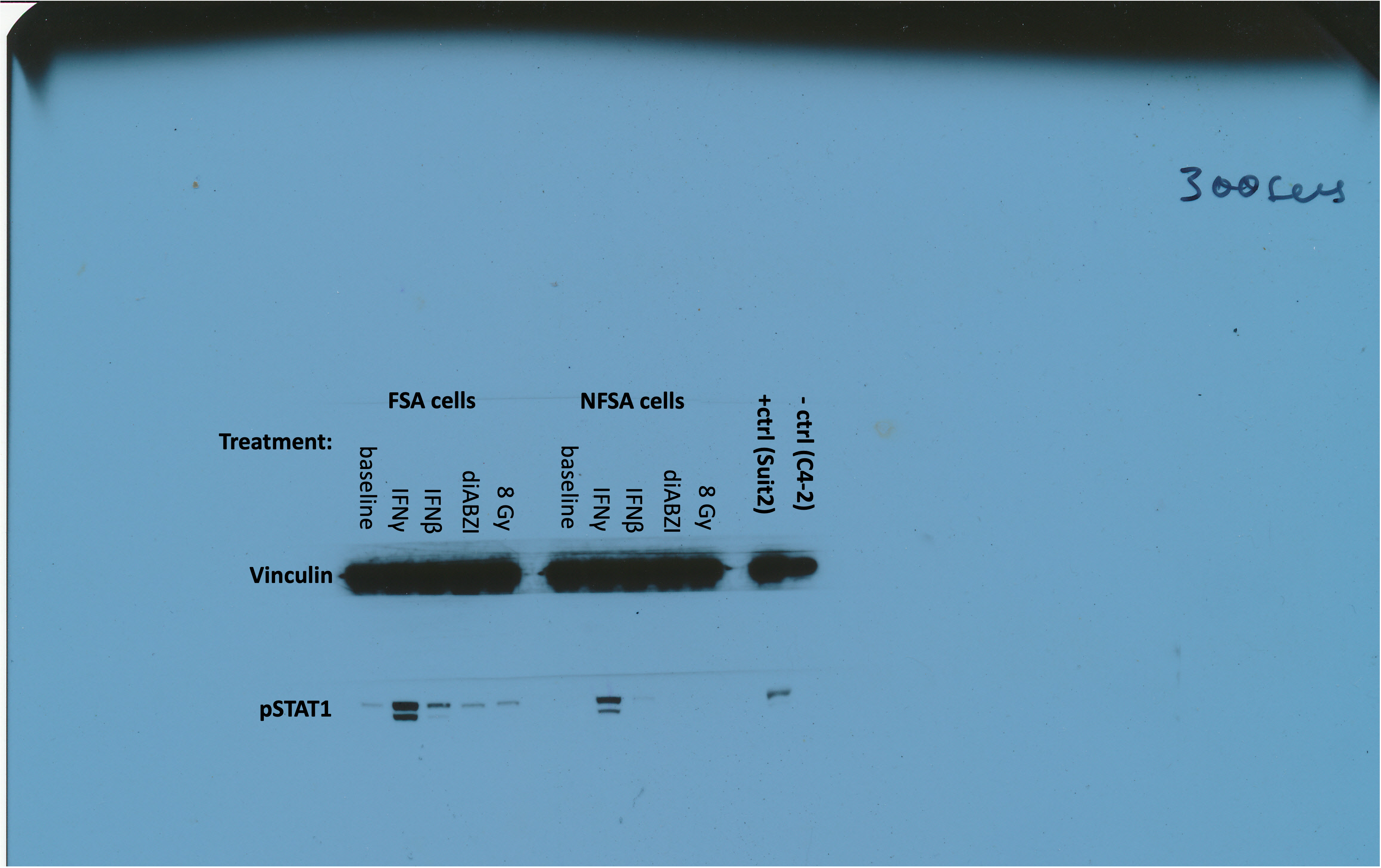

Supplement: Supplementary file 3 — Supplementary Material 4 [file 259_2024_6809_MOESM4_ESM.png]
